# Exaggeration

**Published:** 1880-07

**Authors:** J. B. Chandler


### EXAGGERATION.

J. B. CHANDLER.

With the thermometer at 90° who wants to write or read on subjects requiring depth of thought? As we sit in the shade, dreaming over the current topics, the mind naturally skims the surface and occassionally seizes upon a floating idea making light food for reflection. Perhaps in cooler moments the faint impression thus made may urge to closer analysis and consequent good.

The ambition to be superlative in all things, which seems to mark this people for its own, presents itself just now to our musing brain, and we note the many adjectives belonging to the language, and numerous others coined from slang, which are constantly used to express the excessive beauty of a bonnet, the unsurpassed excellence of a particular doctor or minister, or the wonderful diminutive properties of some individual or pug dog; also the misuse of similar parts of speech, as the *beastly* state of the weather, the *sharpest* girl, or the *tightest* little foot. Now, as the superlative degree can be used truthfully only to one individual or species of a kind, then its misuse involves another besetting sin approaching falsehood, viz: Exaggeration.

Yet, still skimming the surface, we find that some good may and has come out of this failing. A fault exists in the abuse of even good things; the exaggeration of a great beneficial factor.

Let us take Medicine as an instance: few will deny that the exaggerated infinitesimals of Hahnemann have acted beneficially in controlling the abusive use of remedies, and thus wrought out a medium course by which the death rate is greatly lessened. Here, then, is certainly a benefit to mankind, and it is more plainly marked by the effects of the practice of some who still cling to ultraisms of the old theories.

In religion we find the same agents at work. The opera house in Philadelphia, capable of seating between three and four thousand persons, was nearly filled by an audience to listen to Ingersoll's lecture "What shall we do to be saved." The thermometer was ranging between 80° and 90°, yet he held his audience for two hours, listening attentively to words attacking, and seemingly intended to break down, the whole fabric of the Christian religion. Much he said was true, because referring to those parts of religious worship which emanated from circumstances and men; perhaps parts which have served in their time some good as well evil, but which now are retained either from accepted custom or to perpetuate power.

Does any one believe that Mr. Ingersoll can crush out the true religion established by God? No ; the vigorous onslaughts of the orator will possibly aid in dispersing a few motes from before the Golden Gates of the impregnable fort, and men will thus see clearer the beautiful. And right here comes the fact that much good may result and constantly does result from what, in our narrow-mindedness, we deem bad.

Yet we would not advocate the attempt to correct prevailing errors by exaggerated antagonism. Our belief is that man's happiness lies in the middle course, in the contentment which arises from not occupying salient points, marks for rivalry and attack.

Let ambition be gratified in the desire to excel in any particular specialty selected, either mechanical or professional. Thus man raises himself to dignity and develops the utmost capability of his work.

Some men in our country seem to pass their lives in regretting that they can not exercise their constitutional right to be President, because they can not be nominated and elected; and thus many good shoemakers, carpenters, tanners, lawyers, etc., are wrecked.—Whereas superiority and contentment in any selected vocation would produce pleasure and happiness not found "beneath a crown."

